# Progressive genetic aberrations detected by comparative genomic hybridization in squamous cell cervical cancer

**DOI:** 10.1054/bjoc.2000.1509

**Published:** 2000-12

**Authors:** D G Allen, D J White, A-M Hutchins, J P Scurry, S N Tabrizi, S M Garland, J E Armes

**Affiliations:** Departments of Gynaecological Oncology, Pathology, Mercy Hospital for Women, East Melbourne, Victoria, 3002, Australia; Molecular Pathology Laboratory, Victorian Breast Cancer Research Consortium and ^5^Department of Pathology, Department of Pathology, Peter MacCallum Cancer Institute, East Melbourne, Victoria, 3002, Australia; Department of Pathology, Royal Women's Hospital, Carlton, Victoria, 3053, Australia

**Keywords:** comparative genomic hybridization, cervix cancer, lymph node metastasis, human papillomavirus

## Abstract

Genetic changes orchestrated by human papillomaviruses are the most important known factors in carcinogenesis of the uterine cervix. However, it is clear that additional genetic events are necessary for tumour progression. We have used comparative genomic hybridization to document non-random chromosomal gains and losses within a subset of 37 cervical carcinomas matched for clinical stage Ib, but with different lymph node status. There were significantly more chromosomal changes in the primary tumours when the lymph nodes were positive for metastases. The most frequent copy number alterations were loss of 3p, 11q, 6q and 10q and gain of 3q. The smallest areas of loss and gain on chromosome 3 were 3p14–22 and 3q24–26. The study identifies progressive DNA copy number changes associated with early-stage invasive cervical cancers with and without lymph node metastases, a factor of potential prognostic and therapeutic value. © 2000 Cancer Research Campaign http://www.bjcancer.com


*British Journal of Cancer* (2000) **83**(12), 1659–1663
© 2000 Cancer Research Campaign
doi: 10.1054/ bjoc.2000.1509, available online at http://www.idealibrary.com on IDEAL®

http://www.bjcancer.com

### Progressive genetic aberrations detected by comparative genomic hybridization in squamous cell cervical cancer

**DG Allen[1], DJ White[2], A-M Hutchins[2], JP Scurry[3], SN Tabrizi[4], SM Garland[4] and JE Armes[2,5]**

Departments of [1]Gynaecological Oncology, and [3]Pathology, Mercy Hospital for Women, East Melbourne, Victoria, 3002, Australia; Molecular Pathology Laboratory, [2]Victorian Breast Cancer Research Consortium and [5]Department of Pathology, Peter MacCallum Cancer Institute, East Melbourne, Victoria, 3002, Australia; [4]Department of Pathology, Royal Women's Hospital, Carlton, Victoria, 3053, Australia

**Summary** Genetic changes orchestrated by human papillomaviruses are the most important known factors in carcinogenesis of the uterine cervix. However, it is clear that additional genetic events are necessary for tumour progression. We have used comparative genomic hybridization to document non-random chromosomal gains and losses within a subset of 37 cervical carcinomas matched for clinical stage Ib, but with different lymph node status. There were significantly more chromosomal changes in the primary tumours when the lymph nodes were positive for metastases. The most frequent copy number alterations were loss of 3p, 11q, 6q and 10q and gain of 3q. The smallest areas of loss and gain on chromosome 3 were 3p14–22 and 3q24–26. The study identifies progressive DNA copy number changes associated with early-stage invasive cervical cancers with and without lymph node metastases, a factor of potential prognostic and therapeutic value. © 2000 Cancer Research Campaign http://www.bjcancer.com

**Keywords:** comparative genomic hybridization; cervix cancer; lymph node metastasis; human papillomavirus

Cervical cancer remains a major health problem amongst women world-wide. Although the incidence is declining in industrialized nations, the disease remains the most common cause of cancer death in developing countries. Infection with oncogenic genotypes of human papillomavirus (HPV) is the single most important known factor in the pathogenesis of cervical cancer and its precursors (zur Hausen, 1976; Liaw et al, 1999). The process of carcinogenesis is dependent on HPV E6 and E7 genes, which bind with the human p53 and Rb tumour suppressor gene products, respectively, inactivating their function in cell cycle regulation (Durst et al, 1987). However, many women have been exposed to HPV without developing premalignant changes or cervical cancer, which emphasizes the probable involvement of other genetic or epigenetic events essential for tumour progression.

Comparative genomic hybridization (CGH) is a powerful tool in the study of cancer that has fast gained recognition for its ability to scan an entire genome for chromosomal losses or gains. This is achieved by measuring DNA copy number changes, which are mapped to chromosome regions. Chromosome regions gained or lost may harbour oncogenes or tumour suppressor genes, respectively. Only a few studies of CGH in pre-invasive and invasive cervical cancer have been published (Heselmeyer et al, 1996, 1997; Aubele et al, 1998; Kirchhoff et al, 1999; Dellas et al, 1999). Cervical intraepithelial neoplasia grade 3 (CIN 3) and invasive cervical carcinomas have shown similar non-random changes, but quantitatively more changes have been seen in invasive carcinomas (Heselmeyer et al, 1996, 1997; Kirchhoff et al, 1999). In a recent study, DNA losses of chromosome 18q and 11p, together with an increased number of overall losses, were associated with poor prognosis in clinical stage 1b invasive cancers in the absence of lymph node metastases (Dellas et al, 1999). In other tumours, such as breast and kidney cancer, a high number of DNA losses have been associated with poor prognosis (Isola et al, 1995; Moch et al, 1996). Thus it is possible that CGH analysis may indicate prognosis in cancers of the cervix, leading to improved planning of patient management.

In this study, we analysed 37 cases of HPV-typed, clinical stage 1b squamous cell carcinomas of the cervix, with a minimum of 5 years follow-up. 20 cases were from women without lymph node metastases and 17 from women with lymph node metastases. The clinical outcome of stage-matched patients with and without lymph node metastases is known to vary (Martinbeau et al, 1982). Hence, we performed CGH analysis of the primary cervical tumours, as well as the matched lymph node metastases, to determine specific gains and losses associated with both lymph node status and survival.

### MATERIALS AND METHODS

Patients attending the Mercy Hospital for Women for FIGO stage 1b squamous cell carcinoma of the cervix and treated with radical hysterectomy and pelvic lymphadenectomy, between 1986 and 1994, were considered for this study. (FIGO 1b is defined as carcinoma confined to the cervix and greater than 7 mm horizontal dimension and/or 5 mm in depth of invasion, with or without regional lymph node metastases. The equivalent classification is T1b; N0; M0 for cases without regional metastases and T1b; N1; M0 for those with regional metastases.) The first 20 patients without lymph node metastases and 17 patients with lymph node metastases were selected from the database. Follow-up data for a

*Received 21 February 2000*
*Received 25 July 2000*
*Accepted 1 August 2000*

*Correspondence to:* DG Allen minimum of 5 years was available for these patients. Other than stage and lymph node metastasis, no other prognostic factors were considered for selection. Clinical details of recurrences and patient status were obtained from the patient's record. Pathologic review was performed and the histologic type and tumour grades were recorded.

### CGH methods

CGH analysis was performed on the primary cervical tumours as well as the lymph nodes containing metastases. Areas of interest (2–3 $mm^2$) were microdissected from methyl green-stained paraffin-embedded, formalin-fixed tissue sections with a 21 gauge needle and placed into proteinase K digestion buffer. Reference control DNAs were extracted from peripheral blood lymphocytes using a Progenome kit (Progen). DOP-PCR was performed on the tissue lysate as previously described (Kuukasjarvi et al, 1997; Telenius et al, 1992). Amplified DNA samples were labelled by nick translation using the Bionick labelling system (Life Technologies). The probe was hybridized to normal metaphase spreads for 3 days at 37˚C. Tumour samples were labelled with Spectrum Red and hybridized with biotin labelled reference DNA, or digoxigenin and hybridized with Spectrum Green labelled reference DNA to control for differences in hybridization by the different fluorochromes. Following washing, biotinylated DNA was detected by streptavidin-conjugated fluorescein isothiocyanate (FITC) (Vector Laboratories) and digoxigenin-labelled DNA by mouse-anti-digoxigenin antibody conjugated to rhodamine (TRITC) (Boehringer-Mannheim). Samples were counterstained with 4,6-diamino-2-phenylindole (DAPI) in phenyldiamine antifade solution. Each CGH experiment included at least one normal lymph node DNA as a negative control. Images were captured using a Grundig FA87 digital video camera mounted on a Zeiss Axioplan2 epifluorescent microscope equipped with single-band excitation filters for each fluorochrome mounted in a computer controlled Ludl filter wheel. Images were analysed with Quips CGH Analysis Software (Vysis Inc, Downers Grove, IL). A gain of DNA sequence copy number was defined by a tumour/reference ratio >1.2 on both hybridizations. A copy number decrease was defined as having a tumour/reference ratio below 0.8 on both standard and inverse hybridizations. In some situations the tumour/reference ratio did not reach these thresholds but clearly showed copy number alteration when compared to normal controls. This may be due to sample heterogeneity or reduced dynamic range of the metaphase spreads. In these situations the definition of a gain was expanded to include situations when a tumour/reference ratio minus 1 SD was greater than 1.0 and for losses where a tumour/reference ratio plus 1 SD was less than 1.0 in both standard and inverse hybridizations. No attempt was made to distinguish between high-level copy number increases (amplifications) of subregions as contrasted to gains of a whole arm.

### HPV methods

HPV assays were performed to type HPV in the primary tumours. HPV DNA was identified from a single slide by polymerase chain reaction (PCR) with L1 consensus primers MY09 and MYI1 used for amplification (Manos et al, 1989). Reactions underwent 40 cycles of amplification (Bauer et al, 1991; Leyton-Henry et al, 1996). DNA from cloned HPV 6, 11, 16, 18, 31 and 33 was used as positive controls and DNA extracted from normal placenta as negative controls. The positive HPV specimens were typed for HPV 6, 11, 16, 18, 31, 33, 35, 39, 45, 51, 52 with type-specific probes (Resnick et al, 1990). Specimen contamination and carry-over were prevented by using positive displacement pipettes, prior aliquoting of all reagents and performing pre- and post-PCR steps in different rooms specifically allocated for PCR. In addition, to avoid cross-contamination during sectioning of the blocks, the microtome blades were changed, tweezers and cutting area were carefully cleaned with xylene and ethanol between each block.

### Statistical analysis

The Fisher's exact test was used to compare proportions from 2 independent samples and McNemar's test used for paired data. The Wilcoxon test was used to test shift of location from 2 independent samples and the Wilcoxon signed-rank test used for paired data. All tests were 2-sided and results with a $P$ value of less than 0.05 were considered statistically significant. The analyses were carried out using StarXact version 4.

### RESULTS

37 patients with squamous cell carcinoma of the cervix, with a mean age of diagnosis of 48 years (range 30 to 68 years), were studied. All the tumours were either histologic grade 2 or 3. Recurrences of cervical cancer were seen in 9 women. Of these, 8 have died of disease, and one patient treated for vaginal vault recurrence remains free of disease. Another woman died after developing a primary lung cancer 6 years after treatment for her cervical cancer and with no cervical recurrence. 3 patients who died from cervix cancer had no lymph node metastases at the time of diagnosis and 5 patients had metastases. The survival times from diagnosis ranged from 12 to 45 months (mean 27 months). There was an 85% 5-year survival rate for the study group with lymph node metastases, and 71% 5-year survival for the group with lymph node metastases.

CGH was successfully performed on 32 primary tumours: 18 of the 20 patients without lymph node metastases and 14 of the 17 patients with metastases. The individual ratio profile of a representative case is seen in Figure 1. Tissue from lymph node metastases was available for CGH analysis in 12 of the 17 patients and was successful in 10 cases. The primary invasive carcinomas had an average of 3.6 copy number changes per case, compared to 4.9 changes per case for lymph node metastases ($P$ = 0.07). The mean number of CGH changes seen in the primary tumours grouped according to lymph node status is shown in Table 1. There were significantly more CGH-detectable aberrations in the group with lymph node metastases compared to the group without ($P$ = 0.005), due to a significant increase in number of both gains and losses ($P$ = 0.02 and $P$ = 0.03, respectively). However, when primary cancers with lymph node metastases were compared to their matched lymph node metastases, there was no statistical difference in gains, losses or overall changes (Table 1).

The most frequent chromosomal copy number changes in all primary invasive cancers are shown in Figure 2. The most frequent losses were on chromosome arm 3p (56%), 11q (47%), 6q (22%) and 10q (16%) and the most frequent gain on 3q (44%). The smallest regions of copy number alteration could be mapped to loss of 3p14–22 and 11q22–24 and gain of 3q24–26. Table 2 shows the 4 chromosome arms most commonly altered in the 32 primary cervical tumours according to lymph node status.

© 2000 Cancer Research Campaign

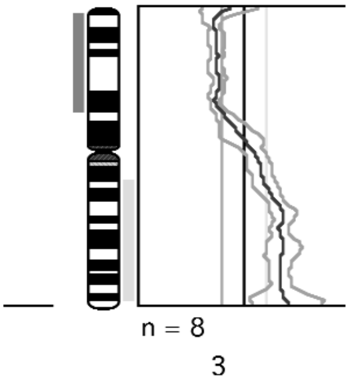

**Figure 1** A CGH ratio profile of a representative primary cervical cancer case. Gains are represented to the right of the midline and to the right of the ideogram and losses to the left of the midline and on the left of the ideogram

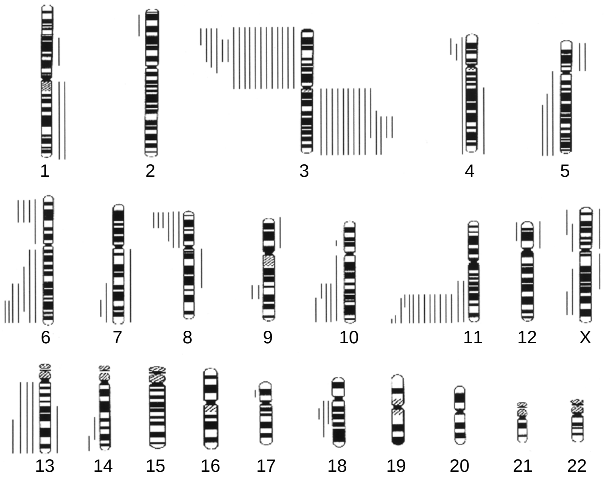

**Figure 2** Chromosome gains (right) and losses (left) in clinical stage 1b primary cervical cancers

**Table 1** Lymph node status and mean CGH changes

| | Gains | Losses | Total changes |
|---|---|---|---|
| Primary tumour; without LN metastases (*n* = 18) | 0.5 | 2.1 | 2.6 |
| Primary tumour; with LN metastases (*n* = 14) | 1.3 | 3.3 | 4.6 |
| LN metastases (*n* = 10) | 0.9 | 4 | 4.9 |

LN = lymph node.

Although not statistically significant to the $P < 0.05$ level, the data show that the occurrence of 3q gain was more frequent in the primary cancers with lymph nodes metastases than those without (64% versus 28%). When the lymph node metastases were matched to the primary tumours, more 11q losses (80% versus 50%) and 6q losses (40% versus 29%) were found in the lymph node metastases although again, this did not reach statistical significance. No trends were noted between the two primary carcinoma groups and the metastatic cancers for 3p loss.

The CGH changes related to survival are shown in Table 3. There were more overall DNA copy number changes in patients who died of disease compared to survivors (4.4 versus 3.3). There were more 3q gains and 3p and 6q losses in those that died of disease. Interestingly, there were more 11q losses in the group that survived. However, the small numbers of non-survivors in the current study precluded definitive correlation between chromosomal copy number changes, sites of recurrent loss or gain and overall survival.

The HPV subtypes were determined in the 32 cervical tumours on which CGH was successfully performed. The majority of tumours were found to have moderate or high-risk subtypes. There was no difference in absolute numbers of CGH detected gains or losses or of chromosome region affected when HPV risk groups were compared.

### DISCUSSION

In this study we used CGH to analyse DNA copy number changes associated with stage 1b cervix cancer with and without lymph node metastases. We also studied the chromosomal alterations within the lymph node metastases. The number of overall chromosomal copy number changes in our series of primary cervical cancers is generally low compared to other cancers (Ried et al, 1994, 1995; Schrock et al, 1994), including our own series of breast cancers (unpublished data). However, the low number of changes is comparable with previous reports of similar stage-matched cervical carcinomas (Heselmeyer et al, 1996; Dellas et al, 1999). There was a significant increase in chromosomal copy number aberrations, including both gains and losses, in the primary cancers with lymph node metastases compared to those without metastases. The number of CGH-detected changes has been shown to increase with stage of cervical cancer and poor prognosis (Heselmeyer et al, 1997; Dellas et al, 1999). This finding is analogous to other cancers, notably breast and kidney, where a high overall number of genetic aberrations is associated with poor prognosis (Isola et al, 1995; Moch et al, 1996). Since patients with stage 1b cervical cancers have a worse prognosis

**Table 2** Common CGH changes in the primary cervical tumour according to lymph node status and in the lymph node metastases

| | 3p loss | 11q loss | 6q loss | 10q loss | 3q gain |
|---|---|---|---|---|---|
| Primary tumours overall (*n* = 32) | 18 (56%) | 15 (47%) | 7 (22%) | 5 (16%) | 14 (44%) |
| Primary tumour; without LN metastases (*n* = 18) | 9 (50%) | 8 (44%) | 3 (17%) | 1 (6%) | 5 (28%) |
| Primary tumour with LN metastases (*n* = 14) | 9 (64%) | 7 (50%) | 4 (29%) | 4 (29%) | 9 (64%) |
| LN metastases (*n* = 10) | 6 (60%) | 8 (80%) | 4 (40%) | 3 (30%) | 7 (70%) |

LN = lymph node.

*© 2000 Cancer Research Campaign*

**Table 3** Patient outcome, mean CGH changes and recurrent CGH changes

| | Gains | Losses | Overall changes | 3q gain | 3p loss | 11q loss | 6q loss |
|---|---|---|---|---|---|---|---|
| Survivors (*n* = 27) | 0.8 | 2.4 | 3.3 | 10 (37%) | 14 (52%) | 14 (52%) | 5 (18%) |
| Non-survivors (*n* = 5) | 1 | 3.4 | 4.4 | 4 (80%) | 4 (80%) | 1 (20%) | 2 (40%) |

when lymph node positive (Martinbeau et al, 1982), the increased number of changes in these cancers indicate that progressive genotypic aberrations are essential to tumour progression, similar to cancers unassociated with virally-induced carcinogenesis.

Several recurrent chromosomal aberrations have been described for cervical cancer, detected either by CGH or allelic imbalance (Hampton et al, 1994; Heselmeyer et al, 1996, 1997; Larson et al, 1997; Kersemaekers et al, 1998; Dellas et al, 1999; Kirchhoff et al, 1999). These regions include gain of 3q and losses of 3p, 11q, and 6q as observed in the current series. Gain of 3q was first reported in the context of cervical cancer as a possible marker for the transition between severe dysplasia and invasive cancer (Heselmeyer et al, 1996). However, a subsequent analysis has indicated that 3q gain is a frequent occurrence even in pre-invasive cancers (Kirchhoff et al, 1999). We show that in our stage-matched invasive cancers, 3q gain was more frequent in the primary cervical tumours with lymph node metastases when compared to those without. Thus it would appear that gain of genetic material on 3q is an important somatic event in cervical oncogenesis, but that its acquisition is not restricted to a specific stage of development. Interestingly, we were able to detect 3q gain more frequently by CGH than in a recent study of similarly-staged cervical cancers (44% versus 15%) (Dellas et al, 1999). However, cancers in this latter study were tumour-enriched rather than microdissected as in the present study, and thus it is possible that 3q gain in carcinoma cells was obscured by contaminating normal cells. In this context it is interesting to note that the same study was able to detect 3q gain as frequently as 56% by FISH analysis, a figure similar to our CGH-detected rate.

Our analysis of cervical cancer was able to localize the smallest area of gain on chromosome 3q to bands 24–26. A previous study has defined the smallest region as 3q26–27 (Heselmeyer et al, 1997), making 3q26 a likely oncogene site important in cervical carcinogenesis. Although there are several named genes mapped to this area, perhaps the human telomerase RNA gene (mapped to 3q26.3) is the most interesting potential oncogene within this region. We detected frequent DNA copy number loss on chromosome arm 3p, which has been previously documented by both CGH and LOH studies (Heselmeyer et al, 1996; Larson et al, 1997; Wistuba et al, 1997; Steenbergen et al, 1998; Kersemaekers et al, 1998; Dellas et al, 1999; Kirchhoff et al, 1999). Loss of 3p appeared with similar frequency in both groups of primary cancers and metastatic cancers. Indeed, this lack of discrepancy between stage 1b primary cancer with and without metastases and metastatic cancers is likely due to an important role of 3p loss early in the pathogenesis of cervical cancer. Consistent with this observation is the finding of frequent deletion of chromosome 3p in pre-invasive cervical cancers detected by LOH analyses (Larson et al, 1997; Wistuba et al, 1997). We were able to define the smallest region of loss on 3p as 14–22. LOH studies have defined 3p14.2 and 3p21 as frequent areas of loss (Wistuba et al, 1997; Kersemaekers et al, 1998). Several tumour suppressor genes have been mapped to this region, including β-catenin. However, this latter gene has effectively been excluded as a significant tumour suppressor gene in cervical cancer (Kersemaekers et al, 1998). Interestingly, a putative telomerase repressor gene has been recently mapped to this region (3p 14.2–p21.1; Tanaka et al, 1998).

Loss of genetic material on 11q was found in 47% of our stage 1b cervical carcinomas. Loss of 11q was also detected by CGH in two other series of early-stage cervical carcinoma (Heselmeyer et al, 1996; Dellas et al, 1999). We were able to map the smallest area of loss to 11q22–24. The identical region has been previously mapped by LOH as a putative site of a tumour suppressor gene important in cervical cancer (Hampton et al, 1994). This region of loss is also involved in carcinogenesis in other malignancies, including breast, colorectal and ovarian cancers and malignant melanoma and this region is known to harbour several candidate tumour suppressor genes (Kersemaekers et al, 1998).

Our study has identified progressive DNA copy number changes associated with early-stage invasive cervical cancers with and without lymph node metastases, a factor of potential prognostic and therapeutic value. In addition, the data contribute significantly to identifying sites of recurrent DNA gain and loss, an important step in the localization of oncogenes and tumour suppressor genes involved in the establishment and progression of cervical cancer.

### ACKNOWLEDGEMENTS

We acknowledge the assistance of Ms Kally Yuen, Statistical Centre, Peter MacCallum Cancer Institute, East Melbourne, Victoria, Australia. This research was funded in part by The Victorian Breast Cancer Research Consortium and the Medical Research Foundation for Women and Babies.

### REFERENCES

Aubele M, Zitzelsberger H, Schenck U, Walch A, Hofler H and Werner M (1998) Distinct cytogenetic alterations in squamous intraepithelial lesions of the cervix revealed by laser-assisted microdissection and comparative genomic hybridization. *Cancer* **84**: 375–379

Bauer HM, Ting Y, Greer CE, Chambers JC, Tashiro CJ, Chimera J, Reingold A and Manos MM (1991) Genital human papillomavirus infection in female university students as determined by a PCR-based method [see comments]. *Jama* **265**: 472–477

Dellas A, Torhorst J, Jiang F, Proffitt J, Schultheiss E, Holzgreve W, Sauter G, Mihatsch MJ and Moch H (1999) Prognostic value of genomic alterations in invasive cervical squamous cell carcinoma of clinical stage IB detected by comparative genomic hybridization. *Cancer Res* **59**: 3475–3479

Durst M, Dzarlieva-Petrusevska RT, Boukamp P, Fusenig NE and Gissmann L (1987) Molecular and cytogenetic analysis of immortalized human primary keratinocytes obtained after transfection with human papillomavirus type 16 DNA. *Oncogene* **1**: 251–6

Hampton GM, Penny LA, Baergen RN, Larson A, Brewer C, Liao S, Busby-Earle RM, Williams, AW, Steel CM, Bird CC and et al (1994) Loss of heterozygosity in

 *© 2000 Cancer Research Campaign*

cervical carcinoma: subchromosomal localization of a putative tumor-suppressor gene to chromosome 11q22–q24. *Proc Natl Acad Sci USA* **91**: 6953–6957

Heselmeyer K, Schrock E, du Manoir S, Blegen H, Shah K, Steinbeck R, Auer G and Ried T (1996) Gain of chromosome 3q defines the transition from severe dysplasia to invasive carcinoma of the uterine cervix. *Proc Natl Acad Sci USA* **93**: 479–484

Heselmeyer K, Macville M, Schrock E, Blegen H, Hellstrom AC, Shah K, Auer G and Ried T (1997) Advanced-stage cervical carcinomas are defined by a recurrent pattern of chromosomal aberrations revealing high genetic instability and a consistent gain of chromosome arm 3q. *Genes Chromosomes Cancer* **19**: 233–240

Isola JJ, Kallioniemi OP, Chu LW, Fuqua SA, Hilsenbeck SG, Osborne CK and Waldman FM (1995) Genetic aberrations detected by comparative genomic hybridization predict outcome in node-negative breast cancer. *Am J Pathol* **147**: 905–911

Kersemaekers AM, Hermans J, Fleuren GJ and van de Vijver MJ (1998) Loss of heterozygosity for defined regions on chromosomes 3, 11 and 17 in carcinomas of the uterine cervix. *Br J Cancer* **77**: 192–200

Kirchhoff M, Rose H, Petersen B, Maahr J, Gerdes T, Lundsteen C, Bryndorf T, Kryger-Baggesen N, Christensen L, Engelholm S and Philip J (1999) Comparative genomic hybridization reveals a recurrent pattern of chromosomal aberrations in severe dysplasia/carcinoma in situ of the cervix and in advanced-stage cervical carcinoma. *Genes, Chromosomes Cancer* **24**: 144–150

Kuukasjarvi T, Tanner M, Pennanen S, Karhu R, Visakorpi T and Isola J (1997) Optimizing DOP-PCR for universal amplification of small DNA samples in comparative genomic hybridization. *Genes Chromosomes Cancer* **18**: 94–101

Larson AA, Liao SY, Stanbridge EJ, Cavenee WK and Hampton GM (1997) Genetic alterations accumulate during cervical tumorigenesis and indicate a common origin for multifocal lesions. *Cancer Res* **57**: 4171–4176

Leyton-Henry J, Scurry JS, Planner RS, Allen D, Sykes P, Garland SM, Borg AJ and Tabrizi SN (1996) Cervical adenoid basal carcinoma, five cases and literature review. *International Journal of Gynecologic Cancer* **6**: 193–199

Liaw KL, Glass AG, Manos MM, Greer CE, Scott DR, Sherman M, Burk RD, Kurman RJ, Wacholder S, Rush BB, Cadell DM, Lawler P, Tabor D and Schiffman M (1999) Detection of human papillomavirus DNA in cytologically normal women and subsequent cervical squamous intraepithelial lesions. *J Natl Cancer Inst* **91**: 954–960

Manos MM TY, Wright DK, Lewis AJ, Broker TR and Wolinsky SM (1989) The use of polymerase chain reaction amplification for the detection of genital human papillomaviruses. *Cancer Cells* **7**: 209–214

Martinbeau P, Kjorstad K and Iversen T (1982) Stage Ib carcinoma of the cervix: the Norwegian Radium Hospital. II. Results when pelvic nodes are involved. *Obstet Gynecol* **60**: 215

Moch H, Presti JC, Jr., Sauter G, Buchholz N, Jordan P, Mihatsch MJ and Waldman FM (1996) Genetic aberrations detected by comparative genomic hybridization are associated with clinical outcome in renal cell carcinoma. *Cancer Res* **56**: 27–30

Resnick RM, Cornelissen MT, Wright DK, Eichinger GH, Fox HS, ter Schegget J and Manos MM (1990) Detection and typing of human papillomavirus in archival cervical cancer specimens by DNA amplification with consensus primers. *J Natl Cancer Inst* **82**: 1477–1484

Ried T, Petersen I, Holtgreve-Grez H, Speicher MR, Schrock E, du Manoir S and Cremer T (1994) Mapping of multiple DNA gains and losses in primary small cell lung carcinomas by comparative genomic hybridization. *Cancer Res* **54**: 1801–1806

Ried T, Just KE, Holtgreve-Grez H, du Manoir S, Speicher MR, Schrock E, Latham C, Blegen H, Zetterberg A, Cremer T and et al (1995) Comparative genomic hybridization of formalin-fixed, paraffin-embedded breast tumors reveals different patterns of chromosomal gains and losses in fibroadenomas and diploid and aneuploid carcinomas. *Cancer Res* **55**: 5415–5423

Schrock E, Thiel G, Lozanova T, du Manoir S, Meffert MC, Jauch A, Speicher MR, Nurnberg P, Vogel S, Janisch W and et al. (1994) Comparative genomic hybridization of human malignant gliomas reveals multiple amplification sites and nonrandom chromosomal gains and losses. *Am J Pathol* **144**: 1203–1218

Steenbergen RD, Hermsen MA, Walboomers JM, Meijer GA, Baak JP, Meijer CJ and Snijders PJ (1998). Non-random allelic losses at 3p, 11p and 13q during HPV-mediated immortalization and concomitant loss of terminal differentiation of human keratinocytes. *Int J Cancer* **76**: 412–417

Tanaka H, Shimizu M, Horikawa I, Kugoh H, Yokota J, Barrett JC and Oshimura M (1998) Evidence for a putative telomerase repressor gene in the 3p14.2–p21.1 region. *Genes Chromosomes Cancer* **23**: 123–133

Telenius H, Carter NP, Bebb CE, Nordenskjold M, Ponder, BA and Tunnacliffe A (1992) Degenerate oligonucleotide-primed PCR: general amplification of target DNA by a single degenerate primer. *Genomics* **13**: 718–725

Wistuba, II, Montellano FD, Milchgrub S, Virmani AK, Behrens C, Chen H, Ahmadian M, Nowak JA, Muller C, Minna JD and Gazdar AF (1997) Deletions of chromosome 3p are frequent and early events in the pathogenesis of uterine cervical carcinoma. *Cancer Res*, **57**: 3154–8

zur Hausen H (1976) Condyloma acuminata and human genital cancer. *Cancer Research*, **36**: 530

*© 2000 Cancer Research Campaign*
